# Bright Single-Photon Source at 1.3 μm Based on InAs Bilayer Quantum Dot in Micropillar

**DOI:** 10.1186/s11671-017-2153-2

**Published:** 2017-05-31

**Authors:** Ze-Sheng Chen, Ben Ma, Xiang-Jun Shang, Hai-Qiao Ni, Jin-Liang Wang, Zhi-Chuan Niu

**Affiliations:** 1 School of Physics and Nuclear Energy Engineering, Beihang University, Beijing, 100191 China; 2 State Key Laboratory for Superlattices and Microstructures, Institute of Semiconductors, Chinese Academy of Sciences, Beijing, 100083 China; 3 College of Materials Science and Opto-Electronic Technology, University of Chinese Academy of Sciences, Beijing, 101408 China; 4 Synergetic Innovation Center of Quantum Information and Quantum Physics, University of Science and Technology of China, Hefei, 230026 Anhui China

**Keywords:** Telecom wavelength, Single-photon source, High emission rate, Micropillar

## Abstract

A pronounced high count rate of single-photon emission at the wavelength of 1.3 μm that is capable of fiber-based quantum communication from InAs/GaAs bilayer quantum dots coupled with a micropillar (diameter ~3 μm) cavity of distributed Bragg reflectors was investigated, whose photon extraction efficiency has achieved 3.3%. Cavity mode and Purcell enhancement have been observed clearly in microphotoluminescence spectra. At the detection end of Hanbury-Brown and Twiss setup, the two avalanched single-photon counting modules record a total count rate of ~62,000/s; the time coincidence counting measurement demonstrates single-photon emission, with the multi-photon emission possibility, i.e., *g* ^2^(0), of only 0.14.

## Background

Optical fiber-based quantum information requires real single-photon sources (SPSs) at telecom band to replace the traditional pseudo-SPSs based on strongly decayed pulse lasers. Self-assembled individual quantum dots (QDs) are potential to emit real single photons and thus have attracted great interest [1–4]. The integration of a distributed Bragg reflector (DBR) cavity to a single QD will enhance its directional emission. Compared to InAs QDs grown on InP substrate emitting at ~1.55 μm with lattice-matched indium-rich materials grown at a low temperature as DBR [5, 6], InAs QDs grown on GaAs substrate are advantageous on the easy integration of lattice-matched high-quality GaAs/Al_0.9_Ga_0.1_As DBR. To realize InAs/GaAs QD SPSs at telecom band, their emission wavelength must extend from the usual one ~0.9 to 1.3 or 1.55 μm and their density must keep as low as 10^7^–10^8^ cm^−2^ to realize single QDs in a microregion. To fabricate low-density InAs QDs by molecular beam epitaxy (MBE), some constructive schemes have been proposed, such as ultralow growth rate [3], high growth temperature [7–9], and precise control of deposition amount [10] of QDs and the isolation of QDs by growth on a mesa/hole-patterned substrate [11] or etching into micropillars [12, 13]. To extend their emission wavelength, several techniques have been developed, such as strain engineering of QDs [14], metamorphic structures [2], and strain-coupled bilayer QD (BQD) structure [15–17]. BQD structure on GaAs substrate is effective to achieve emission above 1.3 μm. High-density BQDs have been applied in laser diodes at ~1.5 μm operating at room temperature [15, 16]. Since it avoids the use of metamorphic layer and ultralow growth rate in the active layer, which might deteriorate the crystal quality [2], the BQD structure is also desired to grow low-density QDs in telecom wavelength. Low-density InAs/GaAs BQDs emitting at 1.3 μm have been obtained in our previous work [18]. To achieve a high count rate of single photons at 1.3 μm for fiber-based applications [2, 19], the photon extraction efficiency from single QDs must be improved. In this letter, by further optimizing the growth conditions of BQD structure and fabricating a micropillar structure, we improve the photon extraction from single InAs/GaAs BQDs emitting at 1.3 μm greatly. The single-photon count rate has reached 62,000 counts/s at the InGaAs single-photon counting module or 3.45 M counts/s at the first objective lens considering the photon collection efficiency of the confocal microscope spectroscopy setup. This is the first time to report a high count rate of single-photon emission at telecommunication wavelength by using InAs/GaAs BQDs. The emission intensity can be further enhanced by introducing an n-type δ-doped layer adjacent to the BQD layer to produce electron charged excitons [13].

## Methods

The investigated sample was grown by solid-source MBE (VEECO Gen930 system) on semi-insulating (100) GaAs substrate. The sample structure consists of, in sequence, a 300-nm-thick GaAs buffer layer, a 25.5-pair wavelength-matched Al_0.9_Ga_0.1_As (113.7 nm)/GaAs (98.6 nm) bottom DBR, a one *λ*-thick undoped GaAs cavity, and an 8-pair Al_0.9_Ga_0.1_As/GaAs upper DBR with the same period. In the center of the GaAs cavity, the active layer for telecom emission, i.e., BQD structure with InGaAs strain-reducing layer, was grown at 470 °C in the Stranski-Krastanov growth mode, which was lower than the temperature used in our previous work. More growth details are reported in Ref. [18]. In this work, specially, micropillar arrays are fabricated on the DBR cavity-coupled BQD samples by photolithography and inductive coupled plasma (ICP) etching with chlorine (Cl_2_) and argon (Ar) mixture gas. As shown in the scanning electron microscope (SEM) image in Fig. 2a, the micropillars are in diameter of ~3 μm and height of 7.75 μm, with very smooth sidewalls. The sample was cooled in a cryogen-free bath cryostat with the temperature finely tuned from 4 to 50 K and excited by a He-Ne laser at wavelength of 633 nm. The confocal microscope setup with an objective (NA, 0.65) focuses the laser into a spot in a diameter of 2 μm and collects the luminescence effectively into a spectrograph, which enables a scanning of microregion to search single QD exciton spectral lines. Microphotoluminescence (μPL) spectrum was detected by a 0.3-m-long focal length monochromator equipped with a liquid-nitrogen-cooled InGaAs linear-array detector for spectrograph. For reflectivity measurement, a spectrophotometer (PerkinElmer 1050) was used with a scanning step of 2 nm and light spot of 3 mm × 3 mm. To investigate the radiative lifetime of the exciton, a time-correlated single-photon counting (TCSPC) board and a Ti:Sapphire pulsed laser (pulse width, ~100 fs; repetition frequency, 80 MHz; wavelength, 740 nm) were used for time-resolved μPL measurement. To measure the second-order autocorrelation function *g* ^(2)^(*τ*), the QD spectral line luminescence was sent to a fiber-coupled Hanbury-Brown and Twiss (HBT) setup [20] and detected by two InGaAs avalanched single-photon counting modules (IDQ 230; time resolution, 200 ps; dark count rate, ~80 counts/s; dead time, 30 μs) and a time coincidence counting module.

## Results and Discussion

**Fig. 1:**
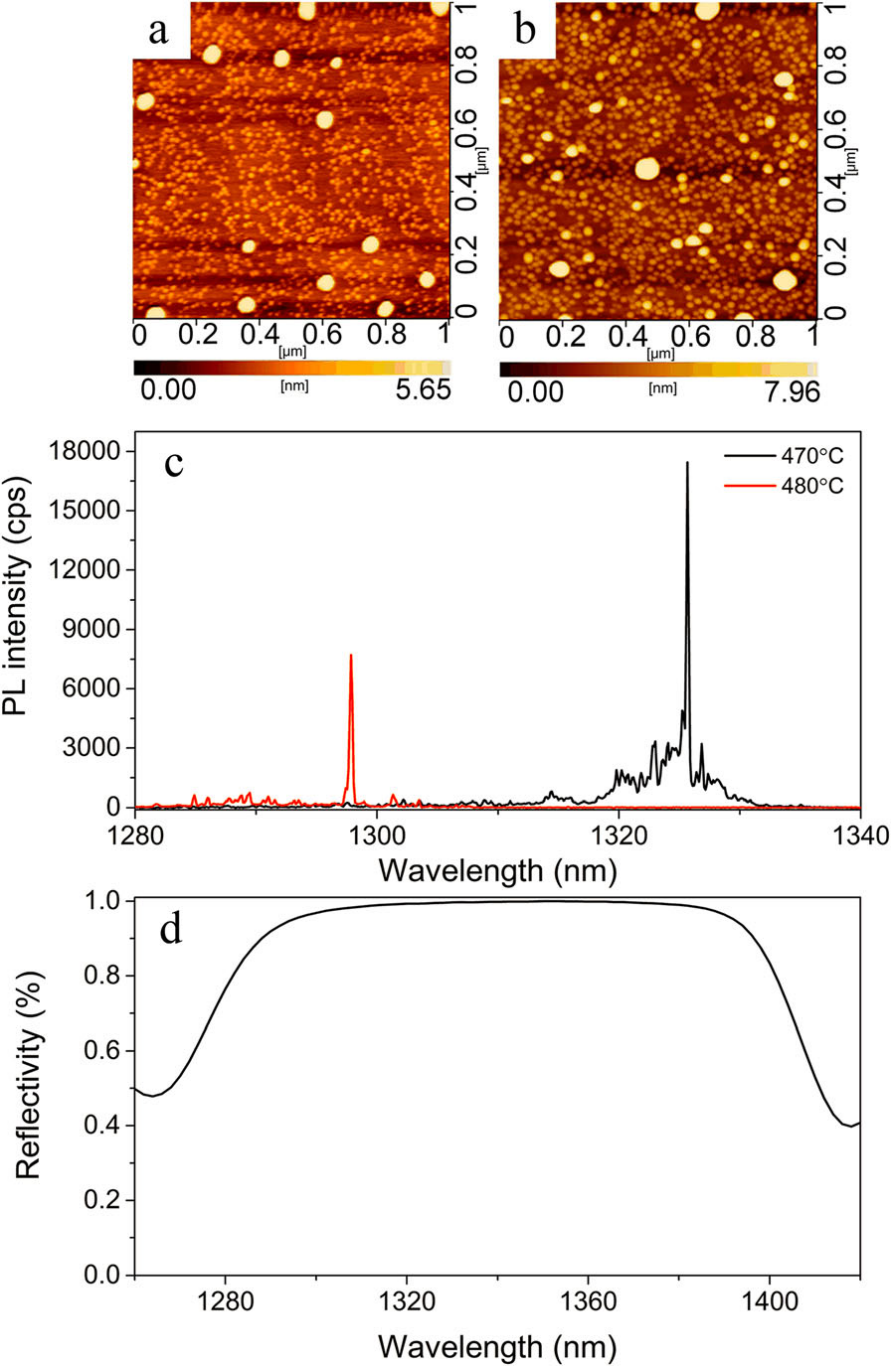
1 × 1 μm^2^ atomic force microscopy (AFM) image of uncapped BQDs grown at **a** 480 and **b** 470 °C. **c** μPL spectra of BQDs embedded in DBR cavities, grown at 480 °C (*red*) and 470 °C (*black*), measured at 4 K. **d** Reflectivity spectrum of the bottom DBR, measured at room temperature

Figure 1a, b shows AFM images of BQDs grown at 480 and 470 °C, respectively. For 480 °C sample, the BQDs are in a mean diameter of 61 nm and a height of about 10 nm. For 470 °C sample, the mean diameter is 75 nm and the height is 13 nm, taller and larger than that grown at 480 °C. The lower temperature contributes to the increased QD size and aspect ratio [21]. To enhance the photon collection efficiency, the BQDs were embedded in a *λ*-thick GaAs cavity and sandwiched between 25.5 lower and 8 upper DBR stacks. All are the same for the two samples, only except the growth temperature of BQDs. As shown in Fig. 1c, the brightest BQDs in the two samples we observed are quite different in PL spectrum. The PL intensity was greatly enhanced at the lower growth temperature, which can be attributed to the reduced strain relaxation and dislocation around BQDs [21]. Figure 1d shows the measured reflectivity spectrum of the bottom DBR, with a value about 99% at a range of 1310–1380 nm, demonstrating a good mirror to reflect QD emission.

**Fig. 2:**
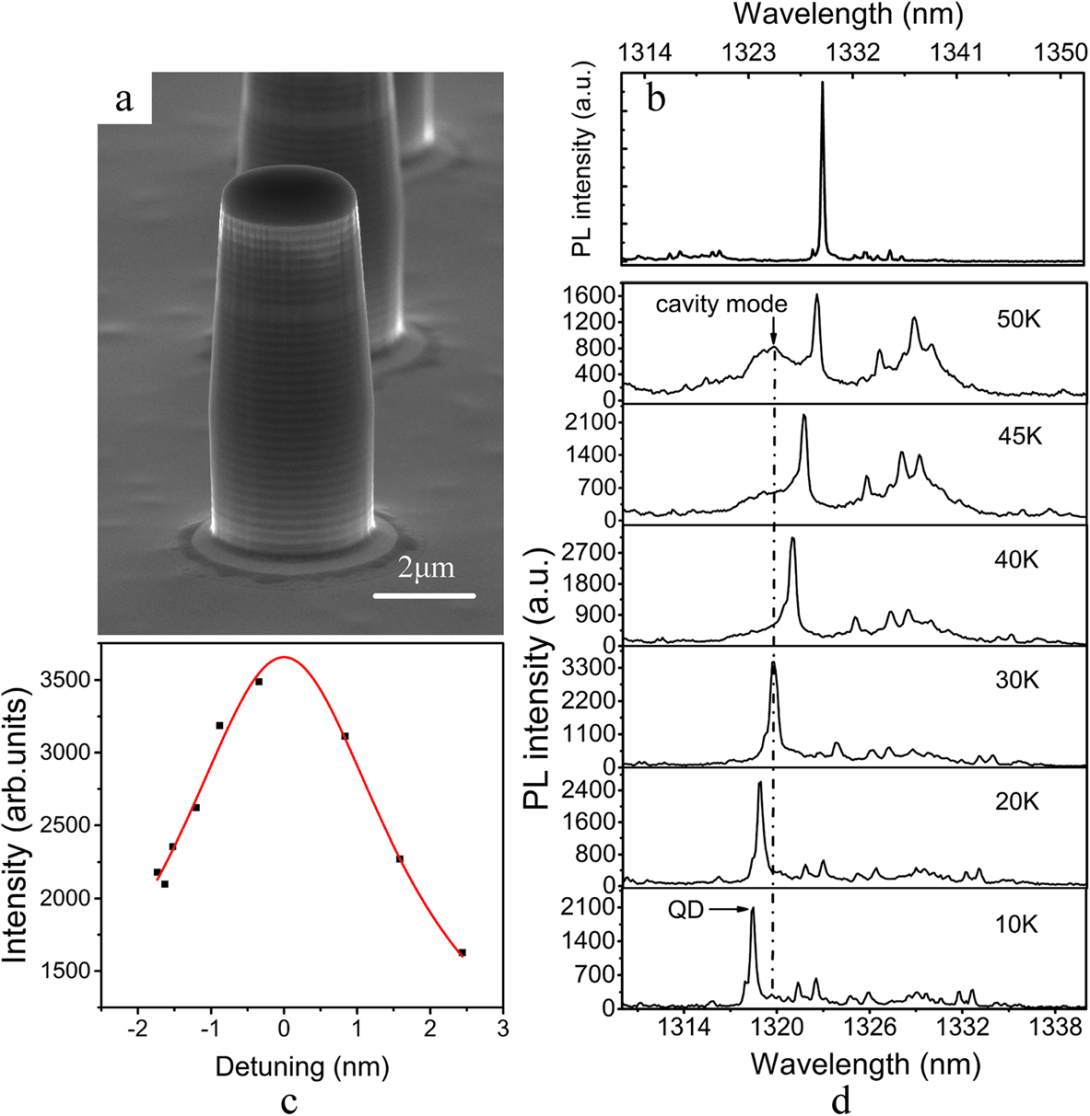
**a** SEM image of the micropillar structure (diameter ~3 μm). **b** Typical PL spectrum of a single BQD in micropillar at 4 K. **d** Temperature-dependent μPL spectra of a typical BQD in micropillar and **c** its integrated PL intensity as a function of the exciton-cavity detuning under excitation power ~2 μW, *red line*: Lorentzian fitting

Figure 2 shows the SEM image of the micropillar and the μPL spectra of a typical BQD embedded in it. Figure 2d shows the μPL spectra as a function of temperature. The emission from the BQD reaches its maximal intensity at 30 K, suggesting a cavity resonance; also see Fig. 2c. The quality factor (Q) of the micropillar cavity is estimated to be about 361. The low Q is attributed to the small reflectivity offset between GaAs and Al_0.9_Ga_0.1_As in the telecom wavelength, and a fewer DBR pairs were used here than the conventional DBRs coupled to QDs emitting at <1 μm [12, 22].

**Fig. 3:**
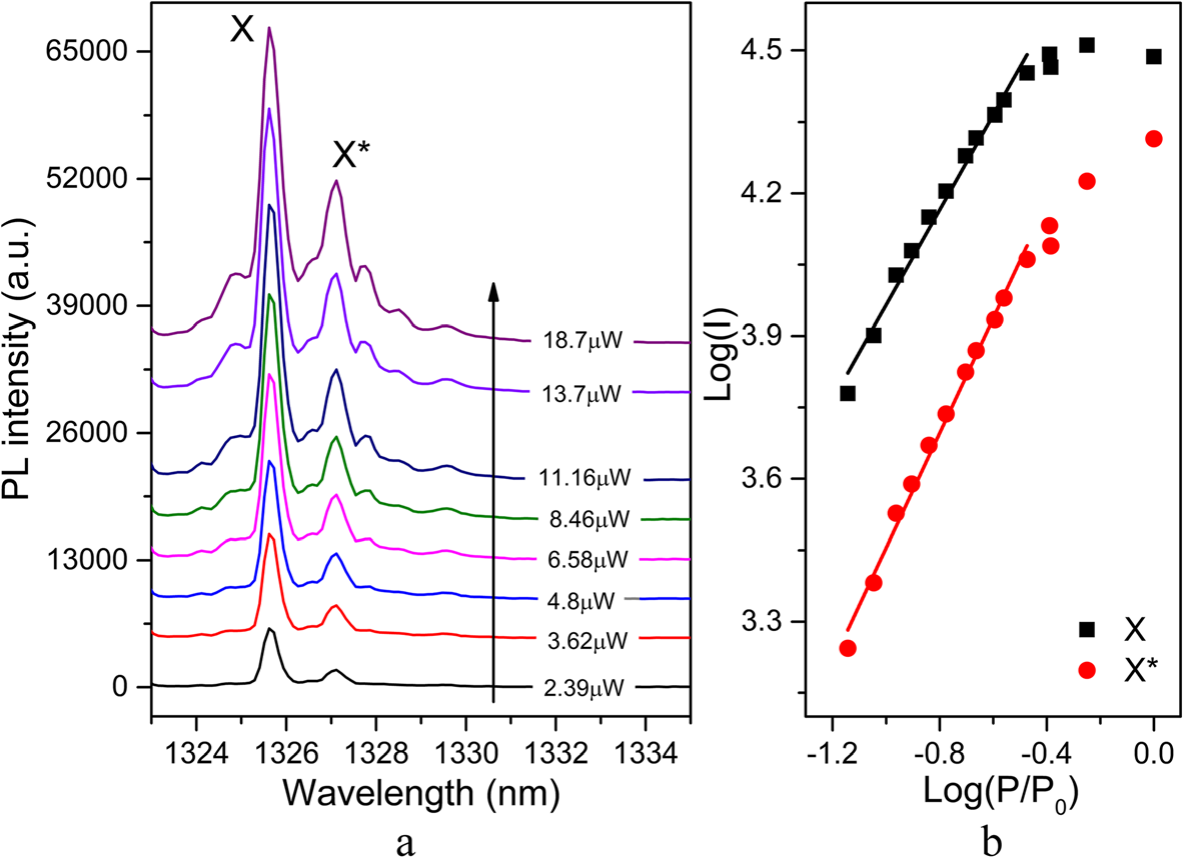
**a** Excitation power-dependent μPL spectra (*T* = 4 K) of typical BQDs in micropillar. **b** Integrated PL intensity of exciton (X) and charged exciton (X*) as a function of excitation power in a log-log scale. *Colored lines*: linear fitting of the experimental data

The excitation power-dependent μPL spectra of InAs/GaAs BQDs in a micropillar was studied by using a continuous wave (cw) He-Ne laser for above-band excitation, as Fig. 3a shows. They show the exciton line (X) at 1325.6 nm and charged exciton line (X*) at 1327.1 nm. The identification of these emission lines is supported by their various power dependences. In Fig. 3b, the integrated PL intensity of X line at 1325.6 nm showed a linear dependence upon the excitation power in the low power region and saturated at a high excitation power. The solid lines are linear fitting to the data in a double-logarithmic plot. The X* line at 1327.1 nm shows a non-saturated excitation power dependence [23]. The followed investigations were performed on the X line.

**Fig. 4:**
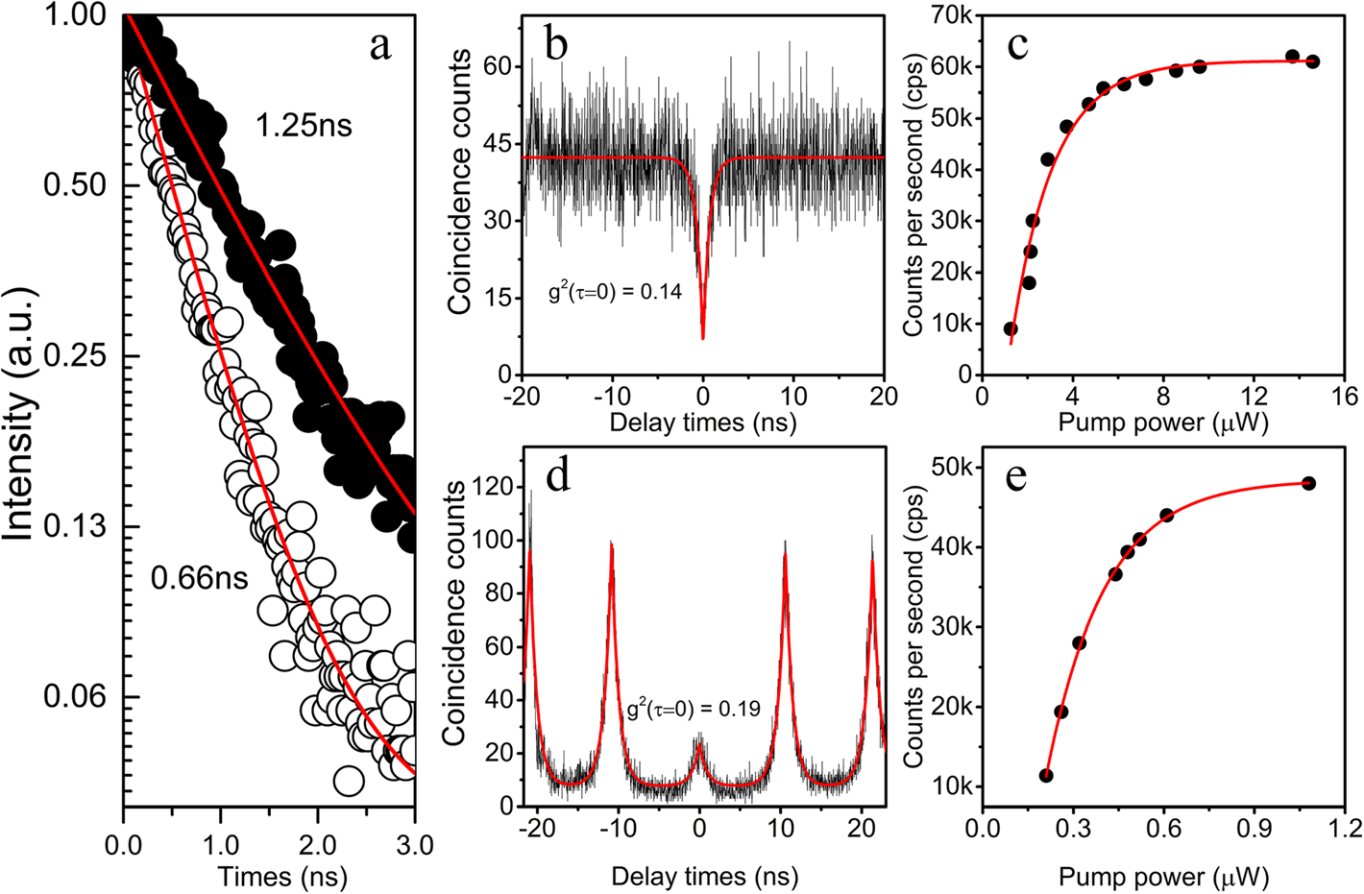
**a** Time-resolved measurements on (*white circle*) and off (black *circle*) resonant of the X line in micropillar, which reveal a Purcell factor of *F*_p_ = 1.9. **b**, **d** Second-order correlation function *g* ^(2)^(*τ*) for the X line under cw excitation and 80 MHz pulse laser excitation at saturated pump power. **c**, **e** Pump power-dependent PL intensity of exciton peak at 1325.6 nm under cw and pulse excitation, respectively. The *black circles* in **c** and **e** denote the count rate recorded at the InGaAs detectors

The time-resolved PL measurements were carried out to determine the Purcell enhancement. The spontaneous emission decay of the BQD X line at QD-cavity resonance and at far detuning are shown in Fig. 4a. The fitted radiative lifetime is 0.66 ns for resonance and 1.25 ns for far detuning, corresponding to a Purcell enhancement factor of 1.9. In order to confirm the single-photon emission of the X line at 1325.6 nm, we measured the second-order correlation function *g* ^(2)^(*τ*) with a HBT setup under cw citation and saturated pulse excitation. Figure 4b shows the measured second-order correlation function of the X line as a function of the delay time *τ* under cw excitation. The data could be fitted with the following expression: *g* ^(2)^(*τ*) = 1 − [1 − *g* ^(2)^(0)]exp(−|*τ*|/*T*) [24]. The fitting results in *g* ^2^(0) = 0.14, proving a single-photon emitter with a strong suppression of the multi-photon emission at zero time delay. The count rate measured on the detectors is presented in Fig. 4c, as a function of the pump power. It shows a linear dependence in the weak pump regime and becomes saturated in the strong pump regime. At saturation, the count rate is around 62,000 counts/s from two InGaAs single-photon detectors, also including the dark counts of the two detectors. To deduce the corresponding number of photons collected in the first lens, we calibrate all the optical loss by using a cw laser at 1320 nm. Transmission loss including microscope objective, long-pass filter, mirrors, and lens and the efficiency of monochromator, lens, and connectors between fibers was 10.46 dB. The detection efficiency and dark count rate of the InGaAs detector with dead times of 30 μs are 18% and ~150 counts/s, respectively. Based on the count rate on InGaAs single-photon detectors and corrected photon count rate by the factor of [1−*g* ^(2)^(0)]^1/2^ [25], we estimate the net single-photon detection rate after compensating the contribution of multi-photon emission and dark count rate is 3.45 × 10^6^ counts/s at the saturated pump power at the first objective lens. To evaluate the photon extraction efficiency of micropillar structure, the measurement under pulsed excitation was also performed. In Fig. 4d, e, we observe a count rate of 48,000/s on the single-photon detectors at the saturated pump power with *g* ^2^(0) = 0.19, under 80 MHz repetition rate laser excitation, which gives a photon extraction efficiency of 3.3% after compensating the contribution of multi-photon emission and considering the efficiency of the detection setup. In our opinion, due to the non-resonant excitation process [12, 26] and low-detection efficiency and long dead time of the InGaAs detector, the observed count rate of single photons may be underestimated.

## Conclusions

In conclusion, we have presented a bright single-photon source at 1325.6 nm by using a single strain-coupled bilayer InAs/GaAs QD in a micropillar Al_0.9_Ga_0.1_As/GaAs DBR cavity. The single-photon emission has really been enhanced by optimizing QD growth temperature and fabricating micropillar structure. The detected single-photon rate reaches 62,000 counts/s, corresponding to a single-photon emission rate of 3.45 MHz at the first objective lens. The photon extraction efficiency is estimated to be about 3.3%, with a Q ~300 micropillar cavity. The second-order autocorrelation measurement with InGaAs single-photon counting modules yielded *g* ^(2)^(0) = 0.14, demonstrating single-photon emission even at high count rate. This is the first time to report so high rate of single-photon emission in the telecom band by using a single InAs/GaAs bilayer QD.

## Abbreviations

AFM: Atomic force microscopy
BQD: Bilayer QD
cw: Continuous wave
DBRs: Distributed Bragg reflectors
HBT: Hanbury-Brown and Twiss
ICP: Inductive coupled plasma
MBE: Molecular beam epitaxy
QDs: Quantum dots
SEM: Scanning electron microscope
SPSs: Single-photon sources
TCSPC: Time-correlated single-photon counting
μPL: Microphotoluminescence
